# Green Synthetic Approaches of 2-Hydrazonothiazol-4(5*H*)-ones Using Sustainable Barium Oxide-Chitosan Nanocomposite Catalyst

**DOI:** 10.3390/polym15183817

**Published:** 2023-09-19

**Authors:** Khaled D. Khalil, Sayed M. Riyadh, Ali H. Bashal, Tariq Z. Abolibda, Sobhi M. Gomha

**Affiliations:** 1 Department of Chemistry, Faculty of Science, Taibah University, Al-Madinah Almunawarah, Yanbu 46423, Saudi Arabia; abishil@taibahu.edu.sa; 2 Department of Chemistry, Faculty of Science, Cairo University, Giza 12613, Egypt; 3 Department of Chemistry, Faculty of Science, Taibah University, Al-Madinah Almunawrah 30002, Saudi Arabia; riyadh1993@hotmail.com; 4 Department of Chemistry, Faculty of Science, Islamic University of Madinah, Madinah 42351, Saudi Arabia; t.z.a@iu.edu.sa (T.Z.A.); s.m.gomha@gmail.com (S.M.G.)

**Keywords:** barium oxide, chitosan, nanocomposite film, heterogenous catalysis, *N*,*N′*-(alkane-diyl)bis(2-chloroacetamide), cyclo-condensation

## Abstract

The diverse applications of metal oxide-biopolymer matrix as a nanocomposite heterogenous catalyst have caused many researches to scrutinize the potential of this framework. In this study, a novel hybrid barium oxide-chitosan nanocomposite was synthesized through a facile and cost-effective co-precipitation method by doping barium oxide nanoparticles within the chitosan matrix at a weight percentage of 20 wt.% BaO-chitosan. A thin film of the novel hybrid material was produced by casting the nanocomposite solution in a petri dish. Several instrumental methods, including Fourier-transform infrared (FTIR), scanning electron microscope (SEM), energy dispersive spectroscopy (EDS), and X-ray diffraction (XRD), were used to analyze and characterize the structure of the BaO-CS nanocomposite. The chemical interaction with barium oxide molecules resulted in a noticeable displacement of the most significant chitosan-specific peaks in the FTIR spectra. When the surface morphology of SEM graphs was analyzed, a dramatic morphological change in the chitosan surface was also discovered; this morphological change can be attributed to the surface adsorption of BaO molecules. Additionally, the patterns of the XRD demonstrated that the crystallinity of the material, chitosan, appears to be enhanced upon interaction with barium oxide molecules with the active sites, OH and NH_2_ groups, along the chitosan backbone. The prepared BaO-CS nanocomposite can be used successfully as an effective heterogenous recyclable catalyst for the reaction of *N*,*N′*-(alkane-diyl)bis(2-chloroacetamide) with 2-(arylidinehydrazine)-1-carbothioamide as a novel synthetic approach to prepare 2-hydrazonothiazol-4(5*H*)-ones. This new method provides a number of benefits, including quick and permissive reaction conditions, better reaction yields, and sustainable catalysts for multiple uses.

## 1. Introduction

Nowadays, researchers are focusing their efforts on manufacturing metal oxide nanoparticles for various applications as interest in nanotechnology grows [1,2,3,4]. Among the processable alkaline earth metal oxides that are used as heterogeneous base catalysts, barium oxide (BaO) is known to be an effective base catalyst for organic transformations such as biodiesel production [5], benzaldehyde reduction [6], transesterification [7], aldol condensation [8], and Tishchenko reaction [9]. Investigations have revealed that this catalyst exhibits the highest catalytic activity. Since barium has the lowest electronegativity among the alkaline earth metals in group 2A, it has the strongest base strength of all alkaline earth metal oxides. The order of the base strengths of oxides of alkaline earth metals is BaO > SrO > CaO > MgO [8,10,11]. Barium oxide (BaO) is a challenging material to prepare, which contributes to the fact that little research has looked at its synthesis and usage as a base catalyst. The conventional process for producing alkaline earth metal oxides involves the thermal breakdown of the suitable hydroxide or carbonate at extremely high temperatures [11]. BaO cannot be properly sampled for use as a solid base catalyst using the traditional preparation method of the thermal breakdown of BaCO_3_ and Ba(OH)_2_ because of its small surface area. Consequently, to address the issue of the high preparation temperatures required for BaO nanoparticles, as well as the drawback of the produced product’s tiny surface area, improvements in nanomaterial production pathways are distinguished by the creation of novel chemicals with privileged physical features.

In recent years, metal oxide nanoparticle stabilization and immobilization have frequently used natural polysaccharides as powerful templates [12,13,14,15]. Due to its distinct structural characteristics, which include the existence of multiple hydroxyl and amino groups, chitosan, the partially deacetylated form of chitin, is thought to be a good stabilizer that can be successfully encased with these metal oxide nanoparticles. Also, this biopolymer material has energy device applications [16,17]. Chitosan is a linear polysaccharide made up of alternating units of glucosamine and *N*-acetylglucosamine. Chitosan contains amino groups that can function as basic catalysts in chemical processes. Recently, chitosan has been utilized as a green and sustainable catalyst that has several advantages over traditional catalysts. Chitosan and its derivatives could be used as green basic promoters in various organic reactions such as Michael additions [18], Aldol condensation [19], Knoevenagel reaction [20], Diels Alder reactions [21], azide-alkyne cycloaddition reactions [22], Suzuki-Miyaura cross-coupling reaction [23], and Sonogashira cross-coupling reaction [24].

Moreover, many researchers have been intrigued by hybrid hydrazonothiazolidin-4-ones owing to their exceptional biological features. Recently, these scaffolds have exhibited antitumor, antimicrobial [25,26], antitubercular [27], anti-Alzheimer, antiviral, and antidepressant activities [28,29], as well as analgesic properties [30]. Also, the constructive nature of hydrazone building blocks has expedited the development of valuable compounds with industrial applications such as preservers, polymers, and glues [31]. 

**Figure 1:**
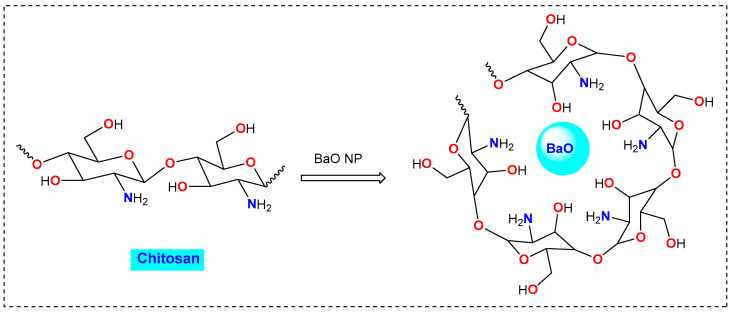
A simplified representation of the barium oxide-chitosan nanocomposite.

With regard to the aforementioned facts and in continuation of our green chemistry research efforts [12,13,14,15], we aimed to create barium oxide (BaO) nanoparticles doped within the matrix of a naturally occurring polysaccharide called chitosan (CS) (Figure 1), and to utilize BaO-CS nanocomposite to promote the synthesis of 2-hydrazonothiazol-4(5*H*)-ones from *N*,*N′*-(alkane-diyl)bis(2-chloroacetamide) via novel synthetic pathway.

## 2. Methods and Materials

### 2.1. Chemicals and Instruments

Barium oxide (powder, CAS No. 1304-28-5; product No. 554847) and chitosan (medium molecular weight, shrimp shells source, batch No. C3646, density = 0.15–0.3 g/cm^3^) were purchased from Sigma-Aldrich (St. Louis, MO, USA). Other substances, such as sodium hydroxide, ethanol, and acetic acid, were bought from the Merck Company (Rahway, NJ, USA) and utilized directly after purchase without additional purification. Thermo Fisher Scientific, Waltham, MA, USA, used a Nicolet Magna 6700 FT spectrometer was used to record Fourier transform infrared (FTIR) spectra in a particular wavenumber range (500–4000 cm^−1^). The electrothermal Gallenkamp apparatus (GallenKamp, Lister, UK) was used to measure the melting points of the isolated products, and these melting points are uncorrected. A Philips diffractometer (Model: X’Pert-Pro MPD; Philips, now PANaytical, Malvern, Worcestershire, UK) was used to examine X-ray diffraction (XRD) patterns using Cu K radiation (wavelength 1.5418 A°) at 40 kV and 40 mA, and the XRD patterns were gathered between 2θ of 5° and 60°, with a 1.5 degree/min scan speed. Thin films were sliced into small parts and adhered to the SEM stubs with carbon tape for SEM and EDX (HRSEM, JSM 6510A, Jeol Ltd., Tokyo, Japan) measurements. The samples were then placed in the SEM Teneo/Quattro for imaging after being coated with a platinum coating of 4 nm thickness where the photos were captured using various magnifications and a high vacuum. The instrument used was SEM Teneo (Thermo Fisher Scientific). 

### 2.2. Preparation of Compounds

#### 2.2.1. Synthesis of Barium Oxide-Chitosan (BaO-CS) Nanocomposite Film

The well-known solution casting method [12,13,14,15] was applied to synthesize barium oxide-chitosan (BaO-CS) nanocomposite film. By dissolving a medium-molecular-weight grade chitosan in the solution for 24 h at room temperature while stirring continuously, we prepared an aqueous acetic acid solution of 2 percent (*w*/*v*). In order to achieve a homogeneous transparent solution, the viscous chitosan solution was filtered using 90 mm Whitman filter paper. The solution was put into a 50 mL bottle, and 20 (*w*/*v*%) barium oxide was gradually added while stirring for 24 h. In order to remove any remaining traces of the solvent, the solution was placed in a Teflon petri dish (8 cm in diameter) and dried for two days in a vacuum oven at 60 °C. After being neutralized with 5 mL of 1 M NaOH and thoroughly washed with distilled water, the BaO-CS nanocomposite film was removed from the petri plate. Following this, the film was stored for two days in a vacuum desiccator at room temperature.

#### 2.2.2. General Procedure for the Preparation of 2-Hydrazonothiazol-4(*5H*)-ones

##### Method A

**Scheme 1:**
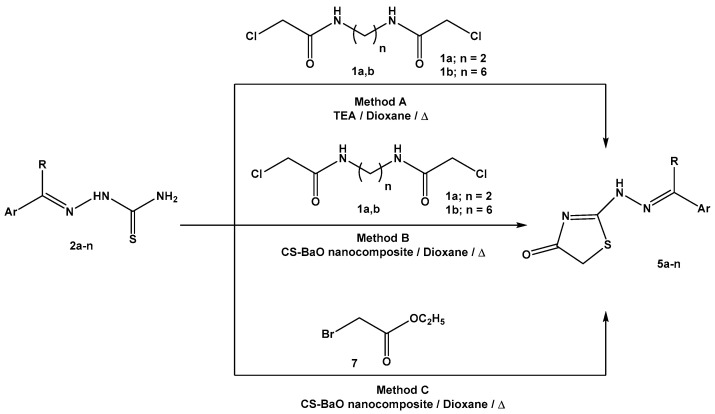
A Schematic diagram for the preparation of 2-hydrazonothiazol-4(5*H*)-ones.

A mixture of *N*,*N′*-(ethane-1,2-diyl)bis(2-chloroacetamide) (**1a**) (0.213 g, 1 mmol) or *N*,*N′*-(hexane-1,6-diyl)bis(2-chloroacetamide) (**1b**) (0.269 g, 1 mmol) and 2-benzylidenehydrazine-1-carbothioamide (**2a**) (2 mmol) was dissolved in 20 mL dioxane containing a few drops of triethylamine. The reaction mixture was thermally heated under constant volume for 3–4 h until all the starting material was consumed (as monitored by TLC). Excess solvent was removed under reduced pressure and the reaction mixture was triturated with methanol. Filtration, washing with methanol, and recrystallization from ethanol are three consecutive processes to obtain the isolated products **5a** (Scheme 1). 

##### Method B

A mixture of (**1a**) (0.213 g, 1 mmol) or (**1b**) (0.269 g, 1 mmol) and suitable 2-(arylidinehydrazine)-1-carbothioamide (**2a**–**n**) (2 mmol) was dissolved in 20 mL dioxane containing 0.1 g of BaO-CS film. The reaction mixture was thermally heated under constant volume for 3–4 h until all the starting material was consumed (as monitored by TLC). Removing the BaO-CS film from the reaction mixture was achieved by filtration and the film was rinsed with hot ethanol once the reaction was completed. Excess solvent was removed under reduced pressure and the reaction mixture was triturated with methanol. Filtration, washing with methanol, and drying are three consecutive processes to obtain the isolated products **5a-n**. Recrystallization of the products was achieved using ethanol (Scheme 1).

##### Method C

2-Benzylidenehydrazine-1-carbothioamide (**2a**) (1 mmol) was dissolved in 20 mL dioxane containing a few drops of triethylamine or 0.1 g of BaO-CS film. Ethyl bromoacetate (**7**) (1 mmol) was added to the solution and the mixture was refluxed and treated as described in Method A and Method B (Scheme 1). 

##### 2-(2-Benzylidenehydrazinyl)thiazol-4(5H)-one (**5a**)

White crystals, mp 253–255 °C [32]; IR (KBr) *υ* = 3200 (NH), 1708 (C=O), 1600 (C=N) cm^−1^; ^1^H NMR (300 MHz, DMSO-*d*_6_) *δ* = 3.85 (s, 2H, S-CH_2_), 7.35–7.99 (m, 5H, Ar-H), 8.21 (s, 1H, CH=N), 11.46 (s, 1H, NH); ^13^C-NMR (DMSO-*d_6_*): *δ* = 33.8 (CH_2_), 127.9, 128.8, 129.7, 131.2, 134.5, 154.8 (Ar-C and C=N), 174.1 (C=O); MS, *m/z* (%) 219 (M^+^, 90). 

##### 2-[2-(4-Methoxybenzylidene)hydrazinyl]thiazol-4(5H)-one (**5b**)

White crystals, mp 129–131 °C [33]; IR (KBr) *υ* = 3214 (NH), 1709 (C=O), 1601 (C=N) cm^−1^; ^1^H NMR (300 MHz, DMSO-*d*_6_) *δ* = 3.76 (s, 3H, OCH_3_), 3.84 (s, 2H, S-CH_2_), 6.99 (d, 2H, Ar-H), 7.67 (d, 2H, Ar-H), 8.30 (s, 1H, CH=N), 11.90 (s, 1H, NH); ^13^C-NMR (DMSO-*d_6_*): *δ* = 33.5 (CH_2_), 55.8 (OCH_3_), 114.8, 127.3, 129.8, 131.2, 156.4, 161.8 (Ar-C and C=N), 174.4 (C=O); MS, *m/z* (%) 249 (M^+^, 70). 

##### 2-[2-(4-Chlorobenzylidene)hydrazinyl]thiazol-4(5H)-one (**5c**)

White crystals, mp 138–139 °C [33]; IR (KBr) *υ* = 3218 (NH), 1708 (C=O), 1599 (C=N) cm^−1^; ^1^H NMR (300 MHz, DMSO-*d*_6_) *δ* = 3.87 (s, 2H, S-CH_2_), 7.49 (d, 2H, Ar-H), 7.74 (d, 2H, Ar-H), 8.38 (s, 1H, CH=N), 12.00 (s, 1H, NH); ^13^C-NMR (DMSO-*d_6_*): *δ* = 33.6 (CH_2_), 128.8, 129.7, 133.5, 135.5, 155.8, 165.9 (Ar-C and C=N), 174.5 (C=O); MS, *m/z* (%) 253 (M^+^, 90). 

##### 2-[2-(4-Bromobenzylidene)hydrazinyl]thiazol-4(5H)-one (**5d**)

White crystals, mp 132–134 °C [33]; IR (KBr) *υ* = 3216 (NH), 1711 (C=O), 1600 (C=N) cm^−1^; ^1^H NMR (300 MHz, DMSO-*d*_6_) *δ* = 3.87 (s, 2H, S-CH_2_), 7.62 (d, 2H, Ar-H), 7.71 (d, 2H, Ar-H), 8.36 (s, 1H, CH=N), 12.00 (s, 1H, NH); MS, *m/z* (%) 298 (M^+^, 85). 

##### 2-[2-(4-Nitrobenzylidene)hydrazinyl]thiazol-4(5H)-one (**5e**)

White crystals, mp 145–146 °C [33]; IR (KBr) *υ* = 3212 (NH), 1710 (C=O), 1601 (C=N) cm^−1^; ^1^H NMR (300 MHz, DMSO-*d*_6_) *δ* = 3.91 (s, 2H, S-CH_2_), 8.02 (d, 2H, Ar-H), 8.17 (d, 2H, Ar-H), 8.25 (s, 1H, CH=N), 11.71 (s, 1H, NH); MS, *m/z* (%) 246 (M^+^, 82). 

##### 2-[2-(4-Hydroxybenzylidene)hydrazinyl]thiazol-4(5H)-one (**5f**)

White crystals, mp 149–151 °C [33]; IR (KBr) *υ* = 3600–3200 (OH & NH), 1708 (C=O), 1600 (C=N) cm^−1^; ^1^H NMR (300 MHz, DMSO-*d*_6_) *δ* = 3.83 (s, 2H, S-CH_2_), 6.81 (d, 2H, Ar-H), 7.56 (d, 2H, Ar-H), 8.24 (s, 1H, CH=N), 10.01 (s, 1H, OH), 11.86 (s, 1H, NH); MS, *m/z* (%) 235 (M^+^, 70). 

##### 2-[2-(3-Hydroxybenzylidene)hydrazinyl]thiazol-4(5H)-one (**5g**)

White crystals, mp 196–198 °C [33]; IR (KBr) *υ* = 3600–3200 (OH & NH), 1703 (C=O), 1599 (C=N) cm^−1^; ^1^H NMR (300 MHz, DMSO-*d*_6_) *δ* = 3.83 (s, 2H, S-CH_2_), 6.81 (s, 1H, Ar-H), 7.56–7.71 (m, 3H, Ar-H), 8.24 (s, 1H, CH=N), 10.02 (s, 1H, OH), 11.87 (s, 1H, NH); MS, *m/z* (%) 235 (M^+^, 72).

##### 2-[2-(Furan-2-ylmethylene)hydrazinyl]thiazol-4(5H)-one (**5h**)

Pale brown powder, mp 233–235 °C [34]; IR (KBr) *υ* = 3221 (NH), 1703 (C=O), 1598 (C=N) cm^−1^; ^1^H NMR (300 MHz, DMSO-*d*_6_) *δ* = 3.86 (s, 2H, S-CH_2_), 6.61–6.98 (m, 2H, furan-H), 7.72–7.86 (m, 1H, furan-H), 8.19 (s, 1H, CH=N), 11.93 (s, 1H, NH); MS, *m/z* (%) 209 (M^+^, 85). 

##### 2-[2-(1-phenylethylidene)hydrazinyl]thiazol-4(5H)-one (**5i**)

Pale orange powder, mp 156–158 °C [34]; IR (KBr) *υ* = 3228 (NH), 1708 (C=O), 1601 (C=N) cm^−1^; ^1^H NMR (300 MHz, DMSO-*d*_6_) *δ* = 2.25 (s, 3H, CH_3_), 3.83 (s, 2H, S-CH_2_), 7.35–7.81 (m, 5H, Ar-H), 11.96 (s, 1H, NH); ^13^C-NMR (DMSO-*d_6_*): *δ* = 15.2 (CH_3_), 32.3 (CH_2_), 126.9, 128.9, 130.3, 138.3, 160.8, 164.6 (Ar-C and C=N), 174.4 (C=O); MS, *m/z* (%) 233 (M^+^, 70). 

##### 2-{2-[1-(4-bromophenyl)ethylidene]hydrazinyl}thiazol-4(5H)-one (**5j**)

Pale brown powder, mp 197–199 °C [35]; IR (KBr) *υ* = 3218 (NH), 1710 (C=O), 1600 (C=N) cm^−1^; ^1^H NMR (300 MHz, DMSO-*d*_6_) *δ* = 2.31 (s, 3H, CH_3_), 3.83 (s, 2H, S-CH_2_), 7.60–7.74 (m, 4H, Ar-H), 11.99 (s, 1H, NH); MS, *m/z* (%) 310 (M^+^, 63). 

##### 2-{2-[1-(thiophen-2-yl)ethylidene]hydrazinyl}thiazol-4(5H)-one (**5k**)

Orange powder, mp 162–164 °C [34]; IR (KBr) *υ* = 3214 (NH), 1709 (C=O), 1599 (C=N) cm^−1^; ^1^H NMR (300 MHz, DMSO-*d*_6_) *δ* = 2.42 (s, 3H, CH_3_), 3.81 (s, 2H, S-CH_2_), 7.04–7.41 (m, 3H, thiophene-H), 11.92 (s, 1H, NH); MS, *m/z* (%) 239 (M^+^, 81). 

2-{2-[1-(pyridin-3-yl)ethylidene]hydrazinyl}thiazol-4(5H)-one (**5l**)

Pale yellow powder, mp 276–278 °C [34]; IR (KBr) *υ* = 3210 (NH), 1712 (C=O), 1600 (C=N) cm^−1^; ^1^H NMR (300 MHz, DMSO-*d*_6_) *δ* = 2.37 (s, 3H, CH_3_), 3.87 (s, 2H, S-CH_2_), 7.58–9.00 (m, 4H, pyridine-H), 12.05 (s, 1H, NH); MS, *m/z* (%) 234 (M^+^, 70). 

##### 2-{2-[1-(1-H-indol-3-yl)ethylidene]hydrazinyl}thiazol-4(5H)-one (**5m**)

Yellow powder, mp 216–218 °C [36]; IR (KBr) υ = 3327, 3237 (2NH), 1714 (C=O), 1600 (C=N) cm^−1^; ^1^H NMR (300 MHz, DMSO-d_6_) δ = 2.46 (s, 3H, CH_3_), 3.85 (s, 2H, S-CH_2_), 7.09–7.42 (m, 5H, indole-H), 11.58 (s, 1H, NH), 11.82 (s, 1H, NH); MS, *m/z* (%) 272 (M^+^, 30). 

##### 2-{2-[1-(2-oxo-2H-chromen-3-yl)ethylidene]hydrazinyl}thiazol-4(5H)-one (**5n**)

Yellow powder, mp 238–240 °C [34]; IR (KBr) *υ* = 3208 (NH), 1738, 1714 (2C=O), 1602 (C=N) cm^−1^; ^1^H NMR (300 MHz, DMSO-*d*_6_) *δ* = 2.46 (s, 3H, CH_3_), 3.84 (s, 2H, S-CH_2_), 7.32–7.80 (m, 5H, coumarin-H), 12.01 (s, 1H, NH); MS, *m/z* (%) 301 (M^+^, 42). 

## 3. Results and Discussion

### 3.1. Structural Characterizations of Nanocatalyst: (BaO-CS) Nanocomposite Film

#### 3.1.1. FTIR-Based Studies 

**Figure 2:**
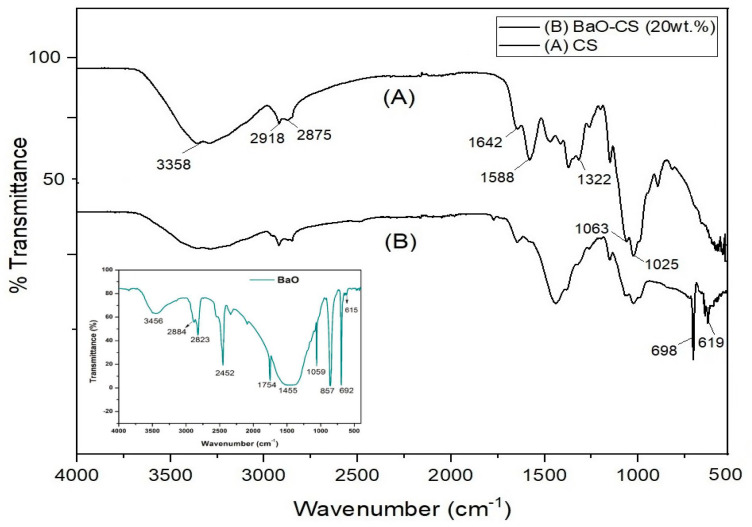
FTIR of (**A**) the native chitosan and (**B**) BaO–CS nanocomposite film (20% wt.).

The following figure, Figure 2, depicts the comparable FTIR analysis for both the original chitosan (A) and the BaO-CS nanocomposite film (B). Due to stretching bands for the H-bonded OH and NH_2_ groups that interfered in the same region of the spectrum (A), this region was seen as a large stretching band at *υ* = 3358 cm^−1^ [12,13,14,15]. Symmetric and asymmetric stretching of the C-H bonds are responsible for the bands produced at around 2918 and 2875 cm^−1^. Additionally, the presence of residual *N*-acetyl groups of chitosan was confirmed by the existence of the characteristic bands which were visible at *υ* = 1642 cm^−1^ (for the C=O, amide stretching band) and 1322 cm^1^ (for the C-N, amide stretching band). The primary amine’s N-H bending is represented by the clear band that first appeared at 1588 cm^−1^. Moreover, the depicted absorption bands at 1063 and 1025 cm^−1^ correspond to C-O stretching bands. Comparably, (B) exhibits the FTIR spectrum of the (BaO-CS) nanocomposite and, particularly in the area around the fingerprint region, there are obvious modifications. As reported in the literature [37], the BaO nanoparticles have characteristic peaks between 600 and 700 cm^−1^ exactly at 619 cm^−1^ and 698 cm^−1^; barium oxide molecules are believed to have been included in these extra peaks, and their coordination with amino and hydroxyl binding sites along the chitosan backbone could explain their appearance. They can be directly attributed to Ba-O bending vibrations.

#### 3.1.2. Characterization via FESEM (Studies on Morphological Features)

**Figure 3:**
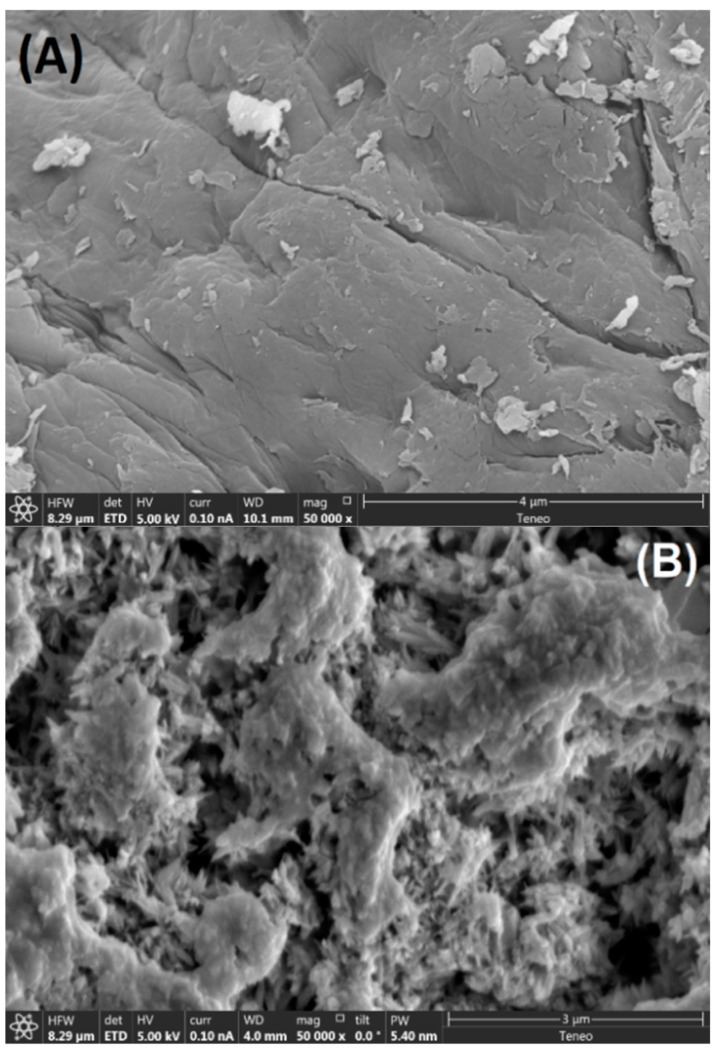
Chitosan (**A**) and 20% wt. barium oxide-chitosan nanocomposite (**B**) from a FESEM study.

FESEM micrographs of chitosan and a composite made of barium oxide and chitosan are shown in Figure 3 to examine the morphological changes induced by the incorporation of BaO molecules within the polymer matrix. As expected, a typical non-porous fibrous surface and smooth-like membranous phase containing dome microfibrils, shaped orifices, and crystallite were examined in the SEM micrograph of chitosan (A), which is in agreement with the literature [12,13,14,15]. In Figure 3B, the BaO-CS composite (B) showed a porous and chain-like-shaped aggregation, and the micrographs clear congregation, homogeneously distributed over the surface of the polymer, has been ascribed to the barium oxide interaction with the chitosan active binding sites (amino and hydroxyl groups). 

#### 3.1.3. Characterization via EDS (Energy Dispersive Spectroscopy)

**Figure 4:**
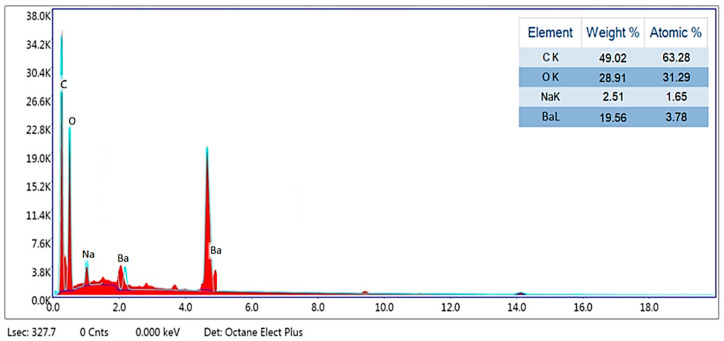
Energy dispersive spectroscopy of BaO-chitosan nanocomposite (20% wt.).

In order to determine the amount of barium present in the chitosan matrix, an EDS graph of a barium oxide-chitosan nanocomposite (20% wt.) was employed in Figure 4. The EDS of the hybrid material revealed the existence of normal Ba signals, indicating that it had been incorporated into the polymer. Figure 4 demonstrates the 19.56%-weighted Ba content.

#### 3.1.4. Characterization via XRD (Crystallinity Studies)

**Figure 5:**
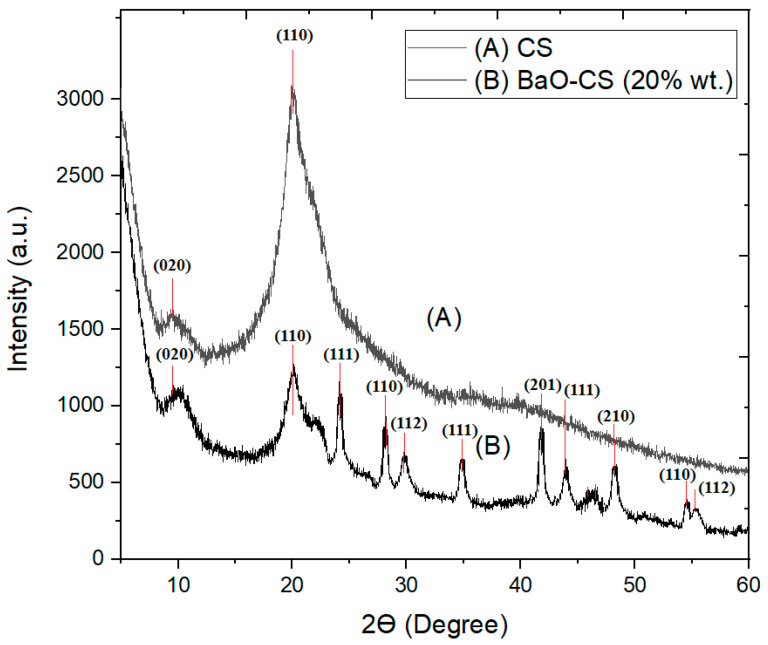
Patterns of XRD of: (**A**) native chitosan and (**B**) barium oxide-chitosan, (20% wt.).

As shown in Figure 5 below, the structural characteristics of the unaltered chitosan (A) and barium oxide-chitosan nanocomposite (20% wt.) (B) were investigated in the 2θ range 0–60°. As shown in Figure 5A, the familiar chitosan peak, which corresponds to the published values for a chitosan-hydrated crystalline structure [12,13,14,15], was clearly observed at 2θ (16–22°). Figure 5B’s XRD pattern shows the same clear peaks of chitosan with a relatively tenuous depression because the barium oxide and chitosan chains were coordinated. BaO’s crystallinity in the nanocomposite was preserved, with just a little shift in the observed peaks, at high 2θ values, compared to what has been documented for commercial BaO. In the XRD spectra of the hybrid material, the additional peaks shown in the diffractogram are caused by the presence of barium oxide molecules and appeared at 2θ = 24.8, 27.2, 29.9, 34.8, 39.9, 42.2, 43.1, 44,8, 46.3, 47.8, 54.9, and 55.1, which conforms to the BaO tetragonal phase and agrees with the previously reported literature [37]. However, the interaction of BaO molecules with the active functional groups (NH_2_ and OH) throughout the chitosan resulted in a little improvement in crystallinity in other locations. Applying the subsequent Debye-Scherrer Formula (1) [38], we obtain:

(1) $$D(nm)=\frac{0.9*\lambda }{\beta .\cos\theta }$$

where *D* (nm) is the size of the crystallite in nm, *β* is the full width at half maximum, (λ) is the Cu-kα1 wavelength = 1.54060 A°, and θ is the diffraction angle. It was calculated that the average crystallite size (*D*) was around 29 nm.

### 3.2. Chemistry

**Scheme 2:**
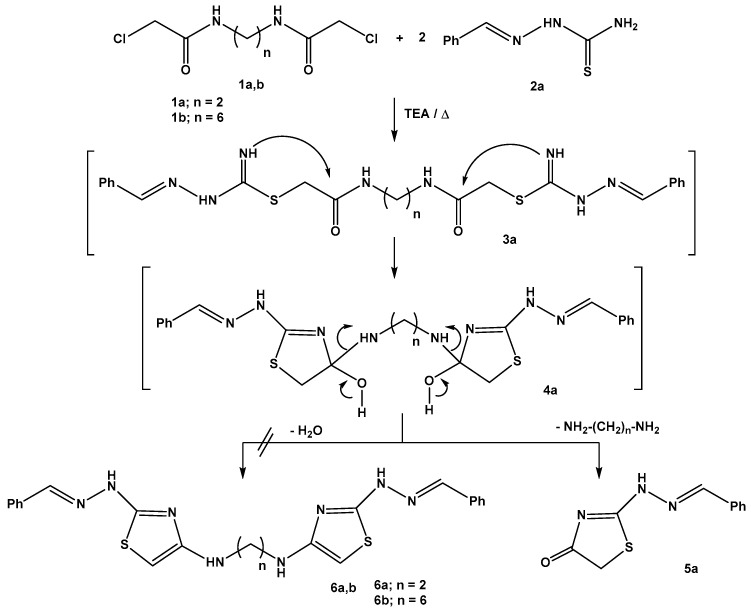
Synthesis of 2-(2-benzylidenehydrazinyl)thiazol-4(5*H*)-one (**5a**).

Initially, two different bis-acetamido derivatives, namely, *N*,*N′*-(ethane-1,2-diyl)bis(2-chloroacetamide) (**1a**) [39] or *N*,*N′*-(hexane-1,6-diyl)bis(2-chloroacetamide) (**1b**) [39] were allowed to react with 2-benzylidenehydrazine-1-carbothioamide (**2a**) (in a molar ratio 1:2) in dioxane and catalytic drops of triethylamine under thermal conditions (Scheme 2). Two synthetic pathways are outlined in Scheme 2. Sequential nucleophilic substitution and intramolecular cyclization afforded the intermediate **4a**. Dehydration of the latter intermediate afforded bis-hydrazonothiazole with the diamino-alkyl linker **6a**. On the other hand, the elimination of alkanediamine from the intermediate **4a** yielded two equivalent moles of 2-hydrazonothiazol-4(5*H*)-one **5a**. Surprisingly, the appearance of a singlet signal of (S-CH_2_) protons at *δ* = 3.90–4.02 ppm [32,40] in the ^1^H-NMR spectra confirmed the formation of two equivalent moles of compound **5a** as an isolated product. Also, the Mass spectrum revealed a molecular ion peak at *m*/*z* 219 corresponding to the molecular formula of compound **5a** (C_10_H_9_N_3_OS).

**Scheme 3:**
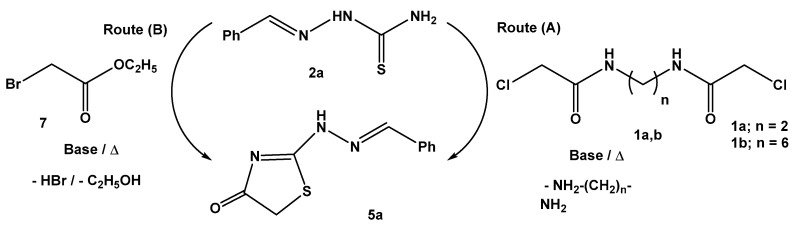
Alternative syntheses of 2-(2-benzylidenehydrazinyl)thiazol-4(5*H*)-one (**5a**).

The key aspect of sustainable methodology in organic synthesis is the development of valuable efficient catalysts. Thus, different synthetic pathways of 2-(2-benzylidenehydrazinyl)thiazol-4(5*H*)-one (5a) were achieved in a comparative study of different basic catalysts (conventional triethylamine and BaO-CS nanocomposite as the green catalyst) and different starting materials (Scheme 3 and Table 1).

As shown in Scheme 3, a variety of cyclo-condensation procedures of 2-benzylidenehydrazine-1-carbothioamide (**2a**) were applied via its reactions with the bis-acetamido derivatives **1a** and **1b** (route A) or ethyl bromoacetate (**7**) (route B). Table 1 reveals that the comparative experiment of the model reaction in route (A) (entries 1–4) demonstrated a significant superiority in yield percentage compared to route (B) (entries 5,6). Also, the heterogenous eco-friendly BaO-CS nanocomposite revealed superior activity over homogenous triethylamine (entries 1–4) due to the ameliorating interaction between the catalyst surface and the reactants. 

Also, the efficiency of the reusability of the BaO-CS nanocomposite was investigated. Thus, in the preparation of compound **5a**, the recovered nanocomposite catalyst was reused three times without a significant loss in its catalytic activity (Table 2).

**Scheme 4:**
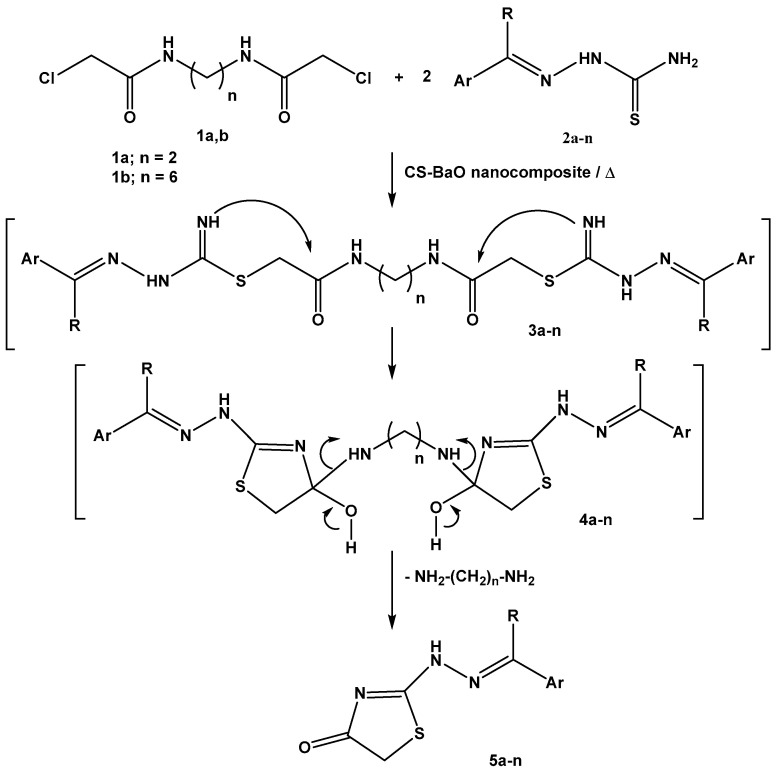
Synthesis of 2-hydrazonothiazolidin-4(5*H*)-ones (**5a**–**n**).

The scope and feasibility of the reactions between the bis-acetamido derivatives **1a** or **1b** and 2-(arylidinehydrazine)-1-carbothioamide **2a**–**n** in dioxane containing 0.1 g of BaO-CS nanocomposite were generalized as shown in Scheme 4 and Table 3.

## 4. Conclusions

In the present study, a hybrid nanocomposite of chitosan doped with barium oxide nanoparticles was synthesized through a facile and cost-effective co-precipitation method at a weight percentage of 20 wt.% BaO/chitosan. The structural elucidation of the nanocomposite film was accomplished through FTIR, XRD, FESEM, and EDS investigations. The chitosan media were found to contain barium oxide nanoparticles, which was confirmed. The FTIR spectra were noticeably different, particularly in the fingerprint area with the distinctive peaks associated with Ba-O bending vibrations. Additionally, the chitosan and BaO characteristic peak pairings were clearly seen in the XRD pattern. The SEM image of the nanocomposite further demonstrated that the surface modification of the chitosan as a result of its coordination with the BaO molecules was distinct and uniform. 

The designed nanocomposite was used successfully as an eco-friendly heterogeneous basic catalyst for a novel synthetic approach to manufacturing 2-hydrazonothiazol-4(5*H*)-one. The gentle reaction conditions, high percentage yield, and use of an affordable, ecologically friendly catalyst are all appealing aspects of this technology, making it a desirable method for extensive industrial applications. 

Additionally, the synthesis of several heterocycles that have previously been produced using hazardous, non-green catalysts like triethylamine, piperidine, or pyridine can be carried out by employing a nanocomposite basic catalyst.

## Figures and Tables

**Table 1:** A comparative yield percentage of compound **5a** via different synthetic pathways.

Entries	Substrates	Catalyst	Yield (%)
**Entry 1**	**2a** + **1a** (molar ratio 2:1)	TEA	85
**Entry 2**	**2a** + **1a** (molar ratio 2:1)	BaO-CS nanocomposite	91
**Entry 3**	**2a** + **1b** (molar ratio 2:1)	TEA	86
**Entry 4**	**2a** + **1b** (molar ratio 2:1)	BaO-CS nanocomposite	92
**Entry 5**	**2a** + **7** (molar ratio 1:1)	TEA	80
**Entry 6**	**2a** + **7** (molar ratio 1:1)	BaO-CS nanocomposite	85

**Table 2:** A comparative yield percentage of compound **5a** via recyclability of BaO-CS nanocomposite.

Entries	Substrates	Fresh Catalyst Yield (%)	Recycled Catalyst		
			(1) Yield (%)	(2) Yield (%)	(3) Yield (%)
**Entry 1**	**2a** + **1a** (molar ratio 2:1)	91	91	89	89
**Entry 2**	**2a** + **1b** (molar ratio 2:1)	92	92	90	90
**Entry 3**	**2a** + **7** (molar ratio 1:1)	85	84	84	83

**Table 3:** Percentage yields of 2-hydrazonothiazolidin-4(5*H*)-ones (**5a**–**n**).

Compd. No.	R	Ar	Yield (%)	Ref.
**5a**	H	C_6_H_5_	92	[36]
**5b**	H	4-CH_3_OC_6_H_4_	91	[37]
**5c**	H	4-ClC_6_H_4_	90	[37]
**5d**	H	4-BrC_6_H_4_	88	[37]
**5e**	H	4-NO_2_C_6_H_4_	89	[37]
**5f**	H	4-HOC_6_H_4_	85	[37]
**5g**	H	3-HOC_6_H_4_	84	[37]
**5h**	H	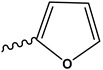		[38]
**5i**	CH_3_	C_6_H_5_	91	[38]
**5j**	CH_3_	4-BrC_6_H_4_	89	[39]
**5k**	CH_3_	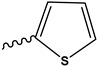	87	[38]
**5l**	CH_3_	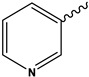	84	[38]
**5m**	CH_3_	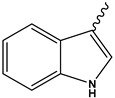	83	[40]
**5n**	CH_3_	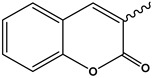	80	[38]

## Data Availability

Data will be available by request.
